# Cholesterol depletion induces transcriptional changes during skeletal muscle differentiation

**DOI:** 10.1186/1471-2164-15-544

**Published:** 2014-06-30

**Authors:** Ana CB Possidonio, Milene Miranda, Gustavo B Gregoracci, Fabiano L Thompson, Manoel L Costa, Claudia Mermelstein

**Affiliations:** Instituto de Ciências Biomédicas, Universidade Federal do Rio de Janeiro, Rio de Janeiro, Brazil; Fundação Oswaldo Cruz, Rio de Janeiro, Brazil; Departamento de Ciências do Mar, Unifesp Baixada Santista, São Paulo, Brazil; Instituto de Biologia, Universidade Federal do Rio de Janeiro, Rio de Janeiro, Brazil

## Abstract

**Background:**

Myoblasts undergo major changes in their plasma membrane during the initial steps of skeletal muscle differentiation, including major alterations in the distribution of cholesterol. Cholesterol is involved in crucial membrane functions, such as fluidity, and permeability, and in the organization of specialized membrane microdomains (or lipid rafts). We have previously shown that alterations in cholesterol levels in myoblasts induce changes in proliferation and differentiation, which involves activation of Wnt/beta-catenin signaling pathway. In this study we used methyl-β-cyclodextrin (MbCD) to extract cholesterol from the membrane of chick skeletal muscle cells grown in culture. Using Ion Torrent-based sequencing, we compared the transcriptome of untreated and MbCD treated cells. Our aim was to define the genes that are expressed in these two conditions and relate their expression to cellular functions.

**Results:**

Over 5.7 million sequences were obtained, representing 671.38 Mb of information. mRNA transcriptome profiling of myogenic cells after cholesterol depletion revealed alterations in transcripts involved in the regulation of apoptosis, focal adhesion, phagosome, tight junction, cell cycle, lysosome, adherens junctions, gap junctions, p53 signaling pathway, endocytosis, autophagy and actin cytoskeleton. Lim domain only protein 7 mRNA was found to be the highest up-regulated feature after cholesterol depletion.

**Conclusions:**

This is the first study on the effects of membrane cholesterol depletion in mRNA expression in myogenic cells. Our data shows that alterations in the availability of plasma membrane cholesterol lead to transcriptional changes in myogenic cells. The knowledge of the genes involved in the cellular response to cholesterol depletion could contribute to our understanding of skeletal muscle differentiation.

**Electronic supplementary material:**

The online version of this article (doi:10.1186/1471-2164-15-544) contains supplementary material, which is available to authorized users.

## Background

During skeletal muscle development, myoblasts undergo a series of cell divisions before they became post mitotic. A number of biochemical and morphological changes occurs in post mitotic myoblasts before their fusion into multinucleated myotubes. These changes include myoblast elongation to a bipolar shape, membrane recognition and alignment, culminating in myoblast fusion. Changes in the composition and structure of the plasma membrane accompany all muscle differentiation steps. One key molecule that regulates the structure and function of the sarcolemma is cholesterol. It has been shown that the addition of cholesterol to the cultured medium before fusion onset inhibits fusion, and that a decrease in membrane cholesterol is necessary for myoblast fusion [1–3]. Using filipin and freeze-fracture electron microscopy, Sekiya and collaborator [4] showed that the early stages of myoblast fusion were characterized by the depletion of cholesterol from the membrane apposition sites, at which the plasma membranes of two adjacent cells were in close contact. Since cholesterol plays an essential role controlling both plasma membrane fluidity and the organization of specialized micro-domains (lipid rafts), it is important to understand its role during myogenesis. One simple approach to study the role of cholesterol during muscle differentiation is to selectively deplete membrane cholesterol from *in vitro* grown myogenic cells. A widely used way of depleting the cholesterol content of cell membranes in a variety of cell types is the incubation of cells with methyl-β-cyclodextrin (MbCD), a compound that has a hydrophobic cavity with a high affinity for cholesterol [5, 6]. Our group has shown that cholesterol depletion by MbCD enhances the fusion of chick-cultured myoblasts and induces the formation of multinucleated myotubes that are more than 3 times thicker than untreated cultures [7]. We also showed that MbCD induces the activation of the Wnt/β-catenin signaling pathway and increases the proliferation of myoblasts [8–10]. However, it is not yet known the genes that are involved in the cellular events that occur after cholesterol depletion of muscle cells. Here, we investigated the effects of membrane cholesterol depletion in the whole transcriptomic profile of chick skeletal muscle cells, using an Ion Torrent-based sequencing. In addition, the morphology of the cholesterol-depleted cells was also evaluated by means of immunofluorescence microscopy.

## Results

### Differences in transcription between untreated and MbCD-treated myogenic cells

To better understand the molecular and cellular basis involved in MbCD-induced muscle differentiation, we analyzed the transcriptome of chick cultured muscle cells after cholesterol depletion. Over 8.5 million sequences were obtained, from which over 5.7 million passed quality control, representing 671.38 mega basepairs of information (Additional file 1: Table S1).

From a total of 4,415 identified transcripts, 1,408 (31.89%) had their transcription significantly modified. Among these, 785 genes were overexpressed in the MbCD-treated myogenic cells compared to the control, indicating up-regulation (Table 1). On the other hand, 623 genes had reduced expression in the MbCD-treated myogenic cells compared to the control, indicating down-regulation (Table 1). The cellular processes that were affected (either down- or up-regulated) after cholesterol depletion were cell growth and death, cell communication, transport and catabolism, and cell motility (Tables 2 and 3).

**Table 1:** **Transcriptomic metadata of chick myogenic cells**

	Transcriptome control	MbCD-treated transcriptome	
**MG-RASTID**	4512803.3	4512804.3	
**Transcriptome Size (post QC) (Mbp)**	337.68	333.70	
**Average size (post QC) (bp)**	115 ± 62	117 ± 58	
**Total number of sequences (post QC)**	2,926,445	2,851,594	
**Classification COG**	499,559 (17.07%)	622,533 (21.83%)	
	**Control**	**MbCD**	**Total**
**Different categories identifiable (COG)**	3,625	4,160	4,415
**Statistically different functions**	n.a.	n.a.	1,408(31.89%)^1^
**Up-regulation with MbCD treatment**	n.a.	785(55.75%)^2^	n.a.
**Down-regulation with MbCD treatment**	623 (44.25%)^2^	n.a.	n.a.

n.a. not applicable, ^1^percentage of total different categories, ^2^percentage of statistically different functions.

From a total of 4,415 identified transcripts, 1,408 (31.89%) had their transcription significantly modified. Among these, 785 genes were up-regulated and 623 were down-regulated after cholesterol depletion.

**Table 2:** **Most abundant down-regulated functions**

COG level 2	COG level 3	COG function	Control relative frequency (%)	MbCD relative frequency (%)	Corrected p-values	Effect size	95.0% lower CI	95.0% upper CI
Cell growth and death	Apoptosis	Interleukin-1 receptor-associated kinase 2	0.012	0.000	5.30E-20	0.012	0.008	0.015
Cell communication	Focal adhesion	Platelet derived growth factor C/D	0.040	0.016	7.68E-14	0.024	0.018	0.031
Transport and catabolism	Phagosome	Rab-interacting lysosomal protein	0.008	0.000	1.73E-13	0.008	0.005	0.011
Cell communication	Tight junction	GTPase KRas	0.008	0.000	1.73E-13	0.008	0.005	0.011
Cell growth and death	Cell cycle	Growth arrest and DNA damage-inducible protein	0.062	0.035	2.54E-09	0.027	0.018	0.035
Cell growth and death	Cell cycle	Regulator of sigma E protease	0.108	0.072	3.14E-09	0.036	0.024	0.048
Cell growth and death	Cell cycle	Proliferating cell nuclear antigen	0.115	0.080	4.06E-08	0.035	0.023	0.047
Transport and catabolism	Lysosome	AP-4 complex subunit mu-1	0.005	0.000	8.67E-08	0.005	0.002	0.007
Cell growth and death	Meiosis	Meiosis induction protein kinase IME2/SME1	0.004	0.000	1.91E-07	0.004	0.002	0.007
Cell growth and death	Cell cycle	RAD24; cell cycle checkpoint protein	0.004	0.000	4.09E-07	0.004	0.002	0.006
Cell communication	Adherens junction	Sorbin and SH3 domain containing 1	0.004	0.000	4.08E-07	0.004	0.002	0.006
Cell communication	Focal adhesion	Fast skeletal myosin light chain 2	0.420	0.356	5.04E-07	0.064	0.041	0.088
Transport and catabolism	Lysosome	cathepsin A (carboxypeptidase C)	0.032	0.016	5.05E-07	0.016	0.010	0.022
Cell communication	Tight junction	Myosin heavy chain	1.108	1.002	5.82E-07	0.105	0.067	0.144
Cell growth and death	Cell cycle	Cyclin-dependent kinase inhibitor 1A	0.039	0.022	1.43E-06	0.017	0.010	0.024
Transport and catabolism	Lysosome	Arylsulfatase B	0.004	0.000	1.90E-06	0.004	0.002	0.006
Cell growth and death	Cell cycle	Cyclin E	0.003	0.000	8.83E-06	0.003	0.001	0.005
Cell communication	Focal adhesion	Actinin alpha	0.363	0.309	9.84E-06	0.053	0.031	0.075
Cell growth and death	Cell cycle	Cell cycle sensor histidine kinase DivJ	0.015	0.006	1.30E-05	0.009	0.005	0.013
Cell communication	Gap junction	Cyclin-dependent kinase 1	0.048	0.030	2.08E-05	0.018	0.010	0.025

**Table 3:** **Most abundant up-regulated functions**

COG level 2	COG level 3	COG function	Control relative frequency (%)	MbCD relative frequency (%)	Corrected p-values	Effect size	95.0% lower CI	95.0% upper CI
Cell communication	Adherens junction	Lim domain only protein 7	0	0.036	3.37E-55	-0.040	-0.041	-0.030
Cell growth and death	Apoptosis	cAMP-dependent protein kinase regulator	0	0.028	4.69E-43	-0.028	-0.032	-0.023
Transport and catabolism	Lysosome	Lysosomal-associated membrane protein 1/2	0	0.025	4.35E-39	-0.025	-0.030	-0.021
Cell growth and death	p53 signaling pathway	Cytochrome c	0	0.023	3.30E-36	-0.023	-0.028	-0.019
Cell growth and death	p53 signaling pathway	BH3 interacting domain death agonist	0	0.021	1.01E-32	-0.021	-0.025	-0.017
Transport and catabolism	Lysosome	CD63 antigen	0	0.021	1.82E-32	-0.021	-0.025	-0.017
Cell growth and death	Cell cycle	Regulatory protein SWI5	0	0.021	3.25E-32	-0.021	-0.025	-0.017
Cell growth and death	Oocyte meiosis	Aurora kinase A	0	0.020	6.84E-31	-0.020	-0.024	-0.016
Transport and catabolism	Lysosome	AP-3 complex subunit delta-1	0	0.019	1.69E-29	-0.019	-0.023	-0.015
Cell communication	Focal adhesion	Classical protein kinase C	0	0.019	9.94E-29	-0.019	-0.022	-0.015
Transport and catabolism	Endocytosis	Hepatocyte growth factor-regulated tyrosine kinase	0	0.018	3.59E-27	-0.018	-0.021	-0.014
Transport and catabolism	Regulation of autophagy	Autophagy-related protein 4	0	0.017	1.24E-26	-0.017	-0.021	-0.014
Transport and catabolism	Phagosome	Vesicle transport protein SEC22	0	0.017	1.58E-25	-0.017	-0.020	-0.013
Cell motility	Regulation of actin cytoskeleton	Bradykinin receptor B2	0	0.017	1.57E-25	-0.017	-0.020	-0.013
Transport and catabolism	Endocytosis	Vacuolar protein sorting-associated protein 45	0	0.015	1.10E-23	-0.015	-0.018	-0.012
Transport and catabolism	Lysosome	AP-1 complex subunit sigma 1/2	0	0.014	1.47E-21	-0.014	-0.017	-0.011
Transport and catabolism	Endocytosis	ESCRT-I complex subunit MVB12	0	0.014	9.84E-21	-0.014	-0.017	-0.010
Cell growth and death	Cell cycle	ATP-dependent Lon protease	0	0.012	7.22E-18	-0.012	-0.015	-0.009
Cell communication	Focal adhesion	p21-activated kinase 1	0	0.011	2.45E-17	-0.012	-0.011	-0.008
Cell communication	Adherens junction	Snail 2	0	0.011	2.43E-17	-0.012	-0.011	-0.008
Transport and catabolism	Endocytosis	E3 ubiquitin-protein ligase NRDP1	0	0.011	3.15E-16	-0.011	-0.014	-0.008
Cell communication	Focal adhesion	Calpain-2	0	0.011	5.87E-16	-0.011	-0.014	-0.008

The group of genes with the highest down-regulation levels after MbCD treatment is involved in apoptosis, focal adhesion, phagosome, tight junction, cell cycle, lysosome, adherens junctions and gap junctions (Table 2). Interleukin-1 receptor-associated kinase 2 mRNA was found to be the highest down-regulated feature (Table 2).

Genes coding for proteins related to adherens junctions, apoptosis, lysosome, p53 signaling pathway, cell cycle, focal adhesion, endocytosis, regulation of autophagy and regulation of actin cytoskeleton were up-regulated after MbCD treatment (Table 3). Lim domain only protein 7 (LMO7) mRNA was found to be the highest up-regulated feature (Table 3). Another highly up-regulated mRNA after cholesterol depletion was the lysosomal-associated membrane protein 1/2 (LAMP 1/2).

### Morphological analysis of cholesterol-depleted myogenic cells

**Figure 1:**
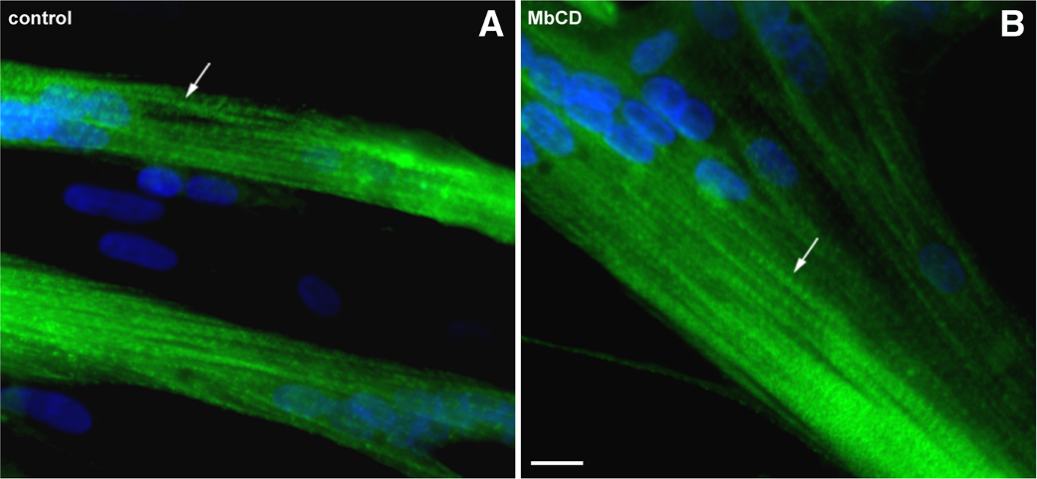
**Methyl-β-cyclodextrin enhances muscle differentiation.** Chick myogenic cells were grown for 24 hours, treated with 2 mM of methyl-β-cyclodextrin (MbCD) for 30 minutes and grown for the next 24 hours. Untreated **(A)** and MbCD-treated cells **(B)** were fixed and double-stained with an anti-sarcomeric α-actinin antibody (green) and the nuclear dye DAPI (blue). Merged images are shown in **A** and **B**. Note the α-actinin distribution in Z-lines along sarcomeres in both untreated and MbCD-treated muscle cells (arrows in **A** and **B**). Scale bar in **B** represents 10 μm.

In order to correlate the transcriptomic analysis with structural characteristics, we analyzed the effects of cholesterol depletion in the morphology of myogenic cultured cells. The effects of MbCD in chick myogenic cultures can be clearly visualized after an immunofluorescence labeling using an antibody against the sarcomeric protein alpha-actinin and the nuclear dye DAPI (Figure 1). Myofibrils labeled with alpha-actinin can be seen in untreated and MbCD-treated cells, but it is noticeable that MbCD treated-cultures displayed a higher number of myofibrils than control cultures (Figure 1). Quantification of the number of myofibrils present within myotubes showed that MbCD treatment induces a 80% increase in the number of myofibrils. The presence of well defined striated myofibrils is an indicator of myogenic differentiation. The presence of much thicker myotubes and a higher number of nuclei within MbCD treated-myotubes as compared to control was also observed (Figure 1). Quantification of myoblast fusion (the number of nuclei in myotubes divided by the total number of nuclei) in cultures grown for 48 hours (untreated and MbCD treated) showed that treatment of myogenic cells with MbCD induces a 15% increase in myoblast fusion. These results showed that MbCD enhances myogenesis by the increase in myoblast fusion which leads to the formation of thicker well-striated myotubes.

**Figure 2:**
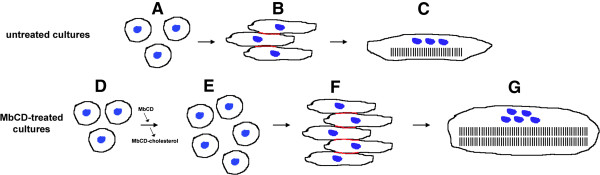
**A proposed model for the effects of MbCD-induced cholesterol depletion in chick myogenic cells.** Our experiments suggest that cholesterol depletion by methyl-β-cyclodextrin (MbCD) enhances myoblast proliferation and its subsequent adhesion and fusion into multinucleated myotubes. Schematically, in normal myogenesis **(A-C)**, myoblasts withdrawal from cell cycle **(A)** and became bipolar myoblasts. These bipolar myoblasts will then align with each other, guided by recognition between their plasma membranes **(B)** and fuse to form long multinucleated myotubes, with sharp striations and aligned nuclei **(C)**. Conversely, myoblasts treated with MbCD **(D)** display an enhancement in cell proliferation **(E)** and fusion **(F)** leading to the formation of thicker myotubes **(G)**. Note that nuclei (shown in blue) are well aligned at the periphery of control myotubes and are clustered in the central region of MbCD-treated myotubes. Areas of membrane adhesion are shown in red.

**Figure 3:**
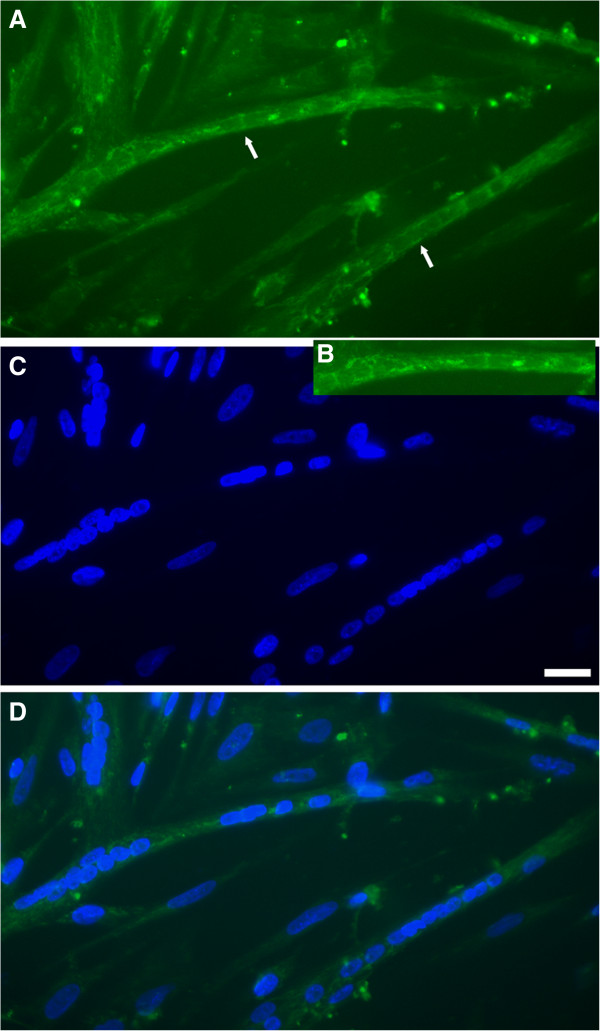
**Lim domain only protein 7 (LMO7) localizes at the nuclei region of chick myotubes.** Chick myogenic cells were grown for 48 hours and double-stained with an anti-LMO7 antibody (green, **A**, **B**, **D**) and the nuclear dye DAPI (blue, **C**, **D**). **B** is an inset of a region of image **A**. A merged image is shown in **D**. Note the strong nuclear envelope staining of the anti-LMO7 antibody in multinucleated myotubes (arrows in **A**), plus a diffuse cytoplasmic fibrillar staining in both mononucleated and multinucleated cells. Scale bar in **C** represents 20 μm.

Interstingly, alpha-actinin mRNA was up-regulated in myogenic cells after MbCD treatment (Additional file 1: Table S1). These transcriptomic results are in accordance with the enhancement in the number of striated myofibrils observed in the immunolabeling for alpha-actinin in MbCD-treated myotubes as compared to untreated cultures (Additional file 1 and Figure 1).A schematic representation of the effects of cholesterol depletion in chick cultures of myogenic cells is shown in Figure 2. Briefly, primary cultures of chick myoblasts treated with MbCD shows an enhancement in cell proliferation and fusion leading to the formation of thicker myotubes, as compared to untreated cultures. Nuclei appear clustered in the central region of MbCD-treated myotubes, while in untreated cultures the nuclei are well aligned at the periphery of myotubes (Figure 2, nuclei in blue). Cholesterol depletion induces the formation of large areas of membrane adhesion in adjacent myoblast prior to cell fusion (Figure 2, red lines). In both control and MbCD-treated cultures it is possible to see the formation of periodic striations in myobrils, but there is a higher number of myofibrils in MbCD treated-myotubes than in control myotubes.Since the Lim domain only protein 7 (LMO7) mRNA was found to be the highest up-regulated feature, we decided to investigate the distribution of LMO7 protein in chick myogenic cells. Immunofluorescence labeling of untreated muscle cell cultures with a polyclonal antibody against LMO7 revealed a strong perinuclear distribution in multinucleated myotubes, plus diffuse cytoplasmic fibrillar localization in both mononucleated and multinucleated cells (Figure 3). No differences were observed in LMO7 distribution in chick myogenic cells after MbCD treatment.

## Discussion

The transcriptomic analysis presented in this study constitutes the first characterization of the genes involved in the response of muscle cells to membrane cholesterol depletion. Our results suggest that the cholesterol depleting drug methyl-β-cyclodextrin induces major changes in the expression of several genes related to apoptosis, focal adhesion, phagosome, tight junction, cell cycle, lysosome, adherens junctions, gap junctions, p53 signaling pathway, endocytosis, regulation of autophagy and regulation of actin cytoskeleton. Some of the induced genes observed in this study code for well-characterized proteins. For instance, Lim domain only protein 7 (LMO7), lysosomal-associated membrane protein 1/2 (LAMP 1/2), hepatocyte growth factor-regulated tyrosine kinase substrate and calpain 2 were all up-regulated in muscle cells after cholesterol depletion.

LMO7 mRNA, which was found to be the highest up-regulated feature, is an emerin-binding protein that regulates the transcription of muscle-specific genes [11]. It has been shown that LMO7 localizes in the nucleus, cytoplasm and cell surface, particularly in cadherin based-adhesion junctions and focal adhesions [12]. Holaska and colleagues speculated that specific signals might release LMO7 from the cell surface, favoring its nuclear localization and enhancing the expression of emerin and other LMO7-dependent genes involved in muscle differentiation. In the present study, we found LMO7 in a perinuclear distribution in multinucleated myotubes, and with diffuse cytoplasmic fibrillar localization in both mononucleated and multinucleated cells. Our group is currently investigating the role of LMO7 during MbCD-induced muscle differentiation. Interestingly, other adhesion proteins, besides LMO7, were found to have their transcription regulated by cholesterol depletion. Adhesion proteins related to adherens junctions, tight junctions, gap junctions and focal adhesion belong to the group of transcripts altered after MbCD treatment (see Tables 2 and 3). Myoblast migration and its subsequent fusion into multinucleated cells are two processes that are highly dependent on cell-cell and cell-extracellular matrix adhesion proteins, and since MbCD enhances myoblast fusion [7], we can hypothesize that the adhesion-related transcripts that were regulated after MbCD are involved in the enhancement of myoblast fusion.

Calpain 2 is one of the adhesion-related mRNA that was up-regulated after cholesterol depletion. Calpain 2, also called m-calpain, is a cytosolic protease, which has been shown to be involved in the membrane reorganization that precedes myoblast fusion [13, 14]. Further, Goudenege and colleagues [15] demonstrated the presence of active m-calpain in myotube caveolae. The increase in the expression of m-calpain after MbCD treatment is in agreement with our previous work showing that cholesterol depletion induces an increase in myoblast fusion [7].

mRNAs that are involved in the regulation of membrane traffic were also altered after cholesterol depletion, as we can observe by the changes in the expression of transcripts related to endocytosis, lysosomes and phagosomes (see Tables 2 and 3). Another was the lysosomal-associated membrane protein 1/2 (LAMP 1/2). LAMP-1 and LAMP-2 are the most abundant glycoproteins of lysosomal membranes, and its mRNAs were found in our study to be highly up-regulated after cholesterol depletion. In embryonic fibroblasts, mutual disruption of both LAMPs is associated with an increased accumulation of autophagic vacuoles, altered lysosomal appearance, and disturbed cholesterol metabolism. It has been shown that unesterified cholesterol accumulates in endo/lysosomal compartments in LAMP double deficient cells [16]. These results suggest a clear connection between the expression and localization of LAMP proteins and cholesterol. The precise role of LAMP proteins during MbCD-induced muscle differentiation needs to be further investigated.

Another transcript involved in the regulation of membrane traffic and which transcription was found altered after MbCD treatment was the hepatocyte growth factor-regulated tyrosine kinase substrate (HGS, HRS, VPS27). It has been reported that galactosylceramide expression factor-1 (GEF-1), a rat homolog of hepatocyte growth factor regulated tyrosine kinase substrate, induces myogenesis in MDCK and C3H10T1/2 cells [17]. Since previous data from our group shows that cholesterol depletion enhances skeletal muscle myogenesis [7–10], we can suggest that HGS is involved in the induction of myogenesis observed in chick myogenic cells after exposure to MbCD.

Interestingly, many cell cycle-related mRNAs were found to have their transcription altered (either up- or down-regulated) after cholesterol depletion. A previous work from our group has shown that cholesterol depletion by MbCD interferes with myoblast proliferation by enhancing the proliferation of chick myogenic cells grown in culture [10]. We found an increase in the levels of p53 expression in MbCD treated-cells when compared to untreated cells [10]. In the present transcriptomic analysis we found the following regulators of cell cycle and p53 signaling pathway altered after MbCD treatment: regulatory protein SWI5, ATP-dependent Lon protease, growth arrest and DNA-damage-inducible protein, regulator of sigma E protease, proliferating cell nuclear antigen, cell cycle checkpoint protein RAD24, cyclin dependent kinase inhibitor 1A, cyclin E, cytochrome c and BH3 interacting domain death agonist.

Importantly, our results show that from a total of 4,415 identified transcripts, 1,408 (31.89%) had their transcription significantly modified in myogenic cells after MbCD treatment. MbCD is widely used as raft-disorganizing agent, since it can remove cholesterol from cell membranes and rafts are highly enriched and dependent on cholesterol. These experiments assume that the major effect of MbCD is the removal of membrane cholesterol. Our work describes a high number of transcripts that have altered (either up- or down-regulated) expression, and therefore care should be taken when using MbCD as a raft-disrupting agent without looking at other possible MbCD-related effects.

## Conclusions

This study analyzed the transcriptome of chick cultured muscle cells after cholesterol depletion. Our data shows that alterations in the availability of plasma membrane cholesterol lead to transcriptional changes in myogenic cells. These results could contribute to the understanding of the role of membrane cholesterol during normal skeletal muscle differentiation, as well as in pathological muscular degenerative disorders.

## Methods

### Antibodies and probes

DNA-binding probe DAPI (4,6-Diamino-2-phenylindole dyhydrochloride) was purchased from Molecular Probes (USA). Rabbit polyclonal anti-desmin antibody, mouse monoclonal anti-sarcomeric α-actinin antibody (clone EA53) and rabbit polyclonal anti-LMO7 antibody were from Sigma-Aldrich (USA). Alexa Fluor 488-goat anti-mouse/rabbit IgG antibodies were from Molecular Probes (USA).

### Primary myogenic cell cultures

This study using chick embryos was approved by the Ethics Committee for Animal Care and Use in Scientific Research from the Federal University of Rio de Janeiro and received the approval number: DAHEICB 004. All cell culture reagents were purchased from Invitrogen (São Paulo, Brazil). Primary cultures of myogenic cells were prepared from breast muscles of 11-day-old chick embryos [7]. Chick embryos were obtained from Granja Tolomei (Rio de Janeiro, Brazil). Cells were plated at an initial density of 2.5 × 10^4^ cells/35 mm culture dishes onto 22 mm-aclar plastic coverslips (Pro-Plastics Inc., USA) previously coated with rat tail collagen. Cells were grown in 2 ml of medium (Minimum Essential Medium with the addition of 10% horse serum, 0.5% chick embryo extract, 1% L-glutamine and 1% penicillin-streptomycin) under humidified 5% CO_2_ atmosphere at 37°C. 24-h cultures were treated for 30 minutes with methyl-β-cyclodextrin (MbCD; Sigma-Aldrich) at a final concentration of 2 mM. The 2 mM final concentration of MbCD was chosen for cell culture treatments because our group has previously shown that 2 mM of MbCD is sufficient to induce skeletal and cardiac muscle cell differentiation without interfering with cell viability [7–10, 18]. After treatment, cultures were washed with fresh cultured medium and grown for the next 24 hours.

The percentage of myoblasts in these chick myogenic cell cultures was calculated by the double-labeling of 24 hour cultures with both DAPI (nuclear staining) and anti-desmin antibody (as a muscle-specific marker) and subsequently counting the number of desmin-positive mononucleated cells out of the total number of cells in the field. On average, myoblasts made up 80% of each culture and non-myogenic cells comprised 20%.

### Immunofluorescence and digital image acquisition

Cells were rinsed with Phosphate Buffered Saline (PBS) and fixed with 4% paraformaldehyde in PBS for 10 min at room temperature. They were then permeabilized with 0.5% Triton-X 100 in PBS 3 times for 10 min. The same solution was used for all subsequent washing steps. Cells were incubated with primary antibodies for 1 h at 37ºC. After incubation, cells were washed for 30 min and incubated with Alexa Fluor-conjugated secondary antibodies for 1 h at 37ºC, and nuclei were labeled with DAPI (0.1 μg/ml in 0.9% NaCl). Cells were mounted in Prolong gold (Molecular Probes, USA) and examined with an Axiovert 100 microscope (Carl Zeiss, Germany). Image processing was performed using Fiji software (based on ImageJ, http://imageJ.nih.gov/ij/). Control experiments with no primary antibodies showed only a faint background staining (data not shown).

### Quantification of fusion index and myofibrils number

Cultures (untreated and MbCD treated) were fixed and labeled with an anti-sarcomeric alpha-actinin antibody and the nuclear dye DAPI. Nuclei (from mononucleated and multinucleated cells) were counted in fifty randomly chosen microscope fields (3 culture dishes, 50 fields in each dish) at a magnification of x 400. The fusion index is defined as the number of nuclei in myotubes divided by the total number of nuclei. A myotube was defined by the presence of at least three nuclei within a continuous cell membrane. The number of myofibrils present within myotubes was counted in fifty randomly chosen microscope fields (3 culture dishes, 50 fields in each dish) at a magnification of x 400.

### Ion Torrent-based cDNA sequencing

The messenger RNA from chick skeletal muscle cells was extracted and isolated by Dynabeads mRNA DIRECT Micro Kit (Invitrogen). Samples were pooled from three biological replicates each. The library preparation was carried out using the Ion Total RNA-Seq Kit v2 (Life Technologies) with 500 ng of PolyA (RNA) according to manufacturer’s instructions. To assess the yield and size distribution of the fragmented RNA we used Qubit RNA Assay Kit (Invitrogen) and Agilent RNA 6000 Pico Kit (Agilent, GE). The complementary DNA (cDNA) was amplified without barcoding. The dsDNA HS Assay Kit (Invitrogen) and Agilent High Sensitivity DNA Kit (Agilent, GE) were used to assess the yield and size distribution of the amplified DNA. The template was made in One Touch System by Ion One Touch 200 template kit V2 (Life technologies) according to manufacturer’s instructions. The transcriptome sequencing was performed using Ion PGM 200 Sequencing Kit (Life Technologies) with 318 chips in Ion Torrent PGM machine.

### Transcriptome analysis

Q20 sequences retrieved from the Ion Browser were submitted to the online server MG-Rast [19] with default Quality Control configuration and to http://www.ensembl.org/Gallus_gallus/Info/Index. QC-passed reads were functionally annotated using the COG database, with minimum e-value of 10^−5^, minimum identity of 60% and minimum alignment length of 15.

### Availability of supporting data

The data sets supporting the results of this article are available in the MG-Rast website repository, through the IDs 4512803.3 (Transcriptome control) and 4512804.3 (MCD-treated). Hyperlink to datasets in: http://metagenomics.anl.gov/linkin.cgi?metagenome=4512803.3http://metagenomics.anl.gov/linkin.cgi?metagenome=4512804.3.

### Statistical analysis

Metagenomic data requires particular statistical analyses, given the nature of the data. The statistical analysis performed was based on the difference between confidence intervals for each category, in each group (control and MbCD treated cells), with proper multiple test correction. Up-regulated transcripts were defined by more abundant expression in treatment than control, while down-regulation was defined by more abundant expression in control than treatment.

Statistical comparison was performed on the freely available Statistical Analysis of Metagenomic Profiles (STAMP) software - version 2.0 [20]. The analysis involved a two-sided hybrid G-test with Yates correction plus an applied Fisher’s exact test; 0.95 confidence intervals were calculated by asymptotic approach with continuity correction and multiple test correction involved Storey’s False Discovery Rate method. P-values less than 0.05 indicated statistical differences.

## Electronic supplementary material

Additional file 1: **Complete transcriptomic analysis of total mRNA expressed in chick cultured muscle cells after cholesterol depletion.** Over 8.5 million sequences were obtained, from which over 5.7 million passed quality control, representing 671.38 mega basepairs of information. (XLS 1 MB)

## References

[1] van der Bosch J, Schudt C, Pette D (1973). Influence of temperature, cholesterol, dipalmitoyllecithin and Ca^2+^ on the rate of muscle cell fusion. Exp Cell Res.

[2] Hirayama E, Sasao N, Yoshimasu S, Kim J (2001). K252a, an indrocarbazole derivative, causes the membrane of myoblasts to enter a fusion-capable state. Biochem Biophys Res Commun.

[3] Nakanishi M, Hirayama E, Kim J (2001). Characterization of myogenic cell membrane: II. Dynamic changes in membrane lipids during the differentiation of mouse C2 myoblast cells. Cell Biol Int.

[4] Sekiya T, Takenawa T, Nozawa Y (1984). Reorganization of membrane cholesterol during membrane fusion in myogenesis *in vitro*: a study using the filipin-cholesterol complex. Cell Struct Funct.

[5] Ohtani Y, Irie T, Uekama K, Fukunaga K, Pitha J (1989). Differential effects of alpha-, beta- and gamma-cyclodextrins on human erythrocytes. Eur J Biochem.

[6] Zidovetzki R, Levitan I (2007). Use of cyclodextrins to manipulate plasma membrane cholesterol content: evidence, misconceptions and control strategies. Biochim Biophys Acta.

[7] Mermelstein CS, Portilho DM, Medeiros RB, Matos AR, Einicker-Lamas M, Tortelote GG, Vieyra A, Costa ML (2005). Cholesterol depletion by methyl-β-cyclodextrin enhances myoblast fusion and induces the formation of myotubes with disorganized nuclei. Cell Tissue Res.

[8] Mermelstein CS, Portilho DM, Mendes FA, Costa ML, Abreu JG (2007). Wnt/beta-catenin pathway activation and myogenic differentiation are induced by cholesterol depletion. Differentiation.

[9] Portilho DM, Martins ER, Costa ML, Mermelstein CS (2007). A soluble and active form of Wnt-3a protein is involved in myogenic differentiation after cholesterol depletion. FEBS Lett.

[10] Portilho DM, Soares CP, Morrot A, Thiago LS, Butler-Browne G, Savino W, Costa ML, Mermelstein C (2012). Cholesterol depletion by methyl-β-cyclodextrin enhances cell proliferation and increases the number of desmin-positive cells in myoblast cultures. Eur J Pharmacol.

[11] Holaska JM, Rais-Bahrami S, Wilson KL (2006). Lmo7 is an emerin-binding protein that regulates the transcription of emerin and many other muscle-relevant genes. Hum Mol Genet.

[12] Ooshio T, Irie K, Morimoto K, Fukuhara A, Imai T, Takai Y (2004). Involvement of LMO7 in the association of two cell-cell adhesion molecules, nectin and E-cadherin, through afadin and alpha-actinin in epithelial cells. J Biol Chem.

[13] Dourdin N, Brustis JJ, Balcerzak D, Elamrani N, Poussard S, Cottin P, Ducastaing A (1997). Myoblast fusion requires fibronectin degradation by exteriorized m-calpain. Exp Cell Res.

[14] Kwak KB, Chung SS, Kim OM, Kang MS, Ha DB, Chung CH (1993). Increase in the level of m-calpain correlates with the elevated cleavage of filamin during myogenic differentiation of embryonic muscle cells. Biochim Biophys Acta.

[15] Goudenege S, Poussard S, Dulong S, Cottin P (2005). Biologically active milli-calpain associated with caveolae is involved in a spatially compartmentalised signalling involving protein kinase C alpha and myristoylated alanine-rich C-kinase substrate (MARCKS). Int J Biochem Cell Biol.

[16] Eskelinen EL, Schmidt CK, Neu S, Willenborg M, Fuertes G, Salvador N, Tanaka Y, Lüllmann-Rauch R, Hartmann D, Heeren J, Von Figura K, Knecht E, Saftig P (2004). Disturbed cholesterol traffic but normal proteolytic function in LAMP-1/LAMP-2 double-deficient fibroblasts. Mol Biol Cell.

[17] Ogura K, Niino YS, Tai T (2004). Galactosylceramide expression factor-1 induces myogenesis in MDCK and C3H10T1/2 cells. Arch Biochem Biophys.

[18] Pontes Soares C, Portilho DM, Da Silva SL, Einicker-Lamas M, Morales MM, Costa ML, Dos Santos MC (2010). Membrane cholesterol depletion by methyl-beta-cyclodextrin enhances the expression of cardiac differentiation markers. Cells Tissues Organs.

[19] Meyer F, Paarmann D, D'Souza M, Olson R, Glass EM, Kubal M, Paczian T, Rodriguez A, Stevens R, Wilke A, Wilkening J, Edwards RA (2008). The metagenomics RAST server - a public resource for the automatic phylogenetic and functional analysis of metagenomes. BMC Bioinformatics.

[20] Parks DH, Beiko RG (2010). Identifying biologically relevant differences between metagenomic communities. Bioinformatics.

